# Neuroscientific results of experimental studies on the control of acute pain with hypnosis and suggested analgesia

**DOI:** 10.3389/fpsyg.2024.1371636

**Published:** 2024-04-04

**Authors:** Wolfgang H. R. Miltner, Marcel Franz, Ewald Naumann

**Affiliations:** ^1^Institute of Psychology, Friedrich Schiller University of Jena, Jena, Thuringia, Germany; ^2^Institute of Psychology, University of Trier, Trier, Rhineland-Palatinate, Germany

**Keywords:** pain, hypnosis, hypnosis analgesia, event-related potentials, fMRI, PET

## Abstract

This narrative review summarizes a representative collection of electrophysiological and imaging studies on the neural processes and brain sources underlying hypnotic trance and the effects of hypnotic suggestions on the processing of experimentally induced painful events. It complements several reviews on the effect of hypnosis on brain processes and structures of chronic pain processing. Based on a summary of previous findings on the neuronal processing of experimentally applied pain stimuli and their effects on neuronal brain structures in healthy subjects, three neurophysiological methods are then presented that examine which of these neuronal processes and structures get demonstrably altered by hypnosis and can thus be interpreted as neuronal signatures of the effect of analgesic suggestions: (A) On a more global neuronal level, these are electrical processes of the brain that can be recorded from the cranial surface of the brain with magnetoencephalography (MEG) and electroencephalography (EEG). (B) On a second level, so-called evoked (EPs) or event-related potentials (ERPs) are discussed, which represent a subset of the brain electrical parameters of the EEG. (C) Thirdly, imaging procedures are summarized that focus on brain structures involved in the processing of pain states and belong to the main imaging procedures of magnetic resonance imaging (MRI/fMRI) and positron emission tomography (PET). Finally, these different approaches are summarized in a discussion, and some research and methodological suggestions are made as to how this research could be improved in the future.

## 1 Biological foundations of pain

Within this narrative review, a representative selection of electrophysiological and imaging studies are summarized, shedding light on the neural processes and brain sources that underlie hypnotic trance, as well as the influence of hypnotic suggestions on the processing of experimentally induced painful stimuli. It complements prior reviews on the impact of hypnosis on brain functions and the structural components related to chronic pain processing (Dillworth et al., 2012; Jensen et al., 2014; Vanhaudenhuyse et al., 2014). It also adds to reviews on a variety of other stimulus modalities and their effect on attentional processes and performance in verbal, numerical, and spatial imagery tasks, on memory, the ability to dissociate and imagine, on depression, anxiety and stress control, and on the ability to calm down and relax (Vanhaudenhuyse et al., 2014; Landry et al., 2017; Wolf et al., 2022).

Despite the enormous public importance, our knowledge of the brain structures involved in pain processing is still evolving and far from complete. Lesion studies, as well as PET and especially MRI/fMRI studies, provide a relatively consistent picture of which structures are essential for processing noxious events to become an impinging event of pain. The first pain processing step occurs in the peripheral nervous system. There, specialized nerve cells, called nociceptors, detect noxious stimuli such as heat, cold, pressure, or tissue damage (Treede, 2016). From these nociceptors, signals travel to the spinal cord, where synapses transmit the information to the thalamus (Bastuji et al., 2016a; Groh et al., 2018), the primary somatosensory (S1) cortex, the secondary somatosensory cortex (S2) and the primary motor cortex (M1). Further information is transmitted to the insular cortex (Segerdahl et al., 2015), the anterior, mid, and post cingulate cortices (Kuner, 2010; Bastuji et al., 2016b; Groh et al., 2018; Del Casale et al., 2022), and to regions in the parietal cortices among them to the precuneus (Bliss et al., 2016). The thalamus also connects to regions of emotion, memory, and fear processing in the amygdala, hippocampus (Tajerian et al., 2018), and subcortical structures including the basal ganglia and brainstem. The thalamus is thus considered the main relay station for sensory information (Sherman, 2016), including noxious signals. From here, further noxious signals are sent to attentional resources that focus on the location of the noxious event in the body schema, and the sensory discriminative aspects of pain and its intensity are processed by the primary and secondary somatosensory cortices S1 and S2 (Bornhovd et al., 2002; Apkarian et al., 2005; Baliki and Apkarian, 2015; Segerdahl et al., 2015). Two other cortical regions, the anterior cingulate cortex (ACC, Iwata et al., 2005) and the insular cortex (ICC, Garcia-Larrea, 2012), are considered components of the classical limbic system and potential candidates for processing the affective-motivational dimension of pain. Under most circumstances, the sensory and affective components of pain are highly correlated, with pain becoming more intense and causing more unpleasant feelings when these structures get activated. Thalamic connections to the secondary somatosensory cortex and the anterior insular cortex, on the other hand, interact with the amygdala to provide an affective evaluation of noxious stimuli (Rainville et al., 2000; Bornhovd et al., 2002; Apkarian et al., 2005; Baliki and Apkarian, 2015; Segerdahl et al., 2015). In addition, connections from the thalamus to frontal motor brain areas and the basal ganglia in the midbrain also prepare and orchestrate appropriate, usually largely automatic escape-avoidance actions (Bastuji et al., 2016b; Corder et al., 2019). The cognitive processing of the pain experience and its embedding into the autobiographical memory is primarily provided by connections to the precuneus as an important hub attentional functions (Rainville et al., 2000; Faymonville et al., 2006), the hippocampal system (Garcia-Larrea and Bastuji, 2018; Tajerian et al., 2018), and to ventromedial and dorsolateral prefrontal cortices (Bromm, 2004). Finally, via connections of the thalamus to the brainstem and medullary regions, the periaqueductal gray (PAG) of the midbrain, the locus coeruleus (Taylor and Westlund, 2017), and the rostral ventral medulla, descending pain control mechanisms can be initiated in addition to appropriate autonomic responses such as the increase of heart rate and blood pressure, further promoting or inhibiting noxious information (Fairhurst et al., 2012; Bastuji et al., 2016a).

The components of this pain processing network interact with each other in a complex way, whereby the activity of the different brain regions changes depending on the type of pain, the intensity of the pain, the emotional state and the autobiographical pain experience of the person, his or her acquired coping skills in dealing with pain, and his or her sociocultural embeddedness (for an extensive summary and time frame of actions in this network see Miltner and Weiss, 1998; Garcia-Larrea and Bastuji, 2018).

## 2 Neurophysiological correlates of pain control

In the following sections of the review, three domains of neurophysiological correlates of pain control will be addressed: (A) On a more global neuronal level, brain electrical processes recorded from the skull surface of the brain with magnetoencephalography (MEG) or electrodes placed across the entire head or electrodes inserted into the skin of the scull using electroencephalography (EEGs). (B) On a second level, so-called evoked (EPs) or event-related potentials (ERPs, Luck, 2014) or event-related magnetic fields (MEFs, Bromm and Scharein, 1996) represent a subgroup of brain electrical parameters of the EEG. (C) The third level of brain research relates to imaging technologies which focus on questions regarding which brain structures are significantly involved in the processing of painful states using imaging methods such as magnetic resonance imaging (MRI/fMRI) or positron emission tomography (PET).

### 2.1 Spontaneous EEG and brain oscillations of pain processing

Pain has been associated with the activation of several spatially distributed and functionally segregated brain structures that serve sensory, cognitive, affective, and motivational aspects of pain processing. Although most of these brain structures are not exclusively activated during pain but also to new events (Mouraux and Iannetti, 2018), the dynamic integration of neuronal responses within these areas is thought to determine the experience of pain. The integration of these processes is ensured by structural connections present within and between brain areas. Since pain can be dynamically modulated by contextual factors and the individual expectation of pain relief as expressed above, the integration of pain-related neuronal processes must be highly flexible. However, this flexibility requires dynamic modifications in neural integration at timescales that cannot be accomplished through alterations in structural connections, but rather through dynamic changes in functional connections (Ploner et al., 2017). It has been suggested that brain oscillations and inter-areal synchronization may be instrumental in neuronal integration and the flexible routing of information flow between distributed brain networks (Bastos et al., 2015; Ploner et al., 2017; Singer, 2018). Brain oscillations represent rhythmic neuronal signals that are ubiquitous in the brain and can be recorded by local field potentials, electroencephalography, and magnetencephalography. While the EEG simultaneously records the brain's electrical voltage fields of thousands of neurons as oscillatory voltage-time diagrams (i.e., changes of polarization and strength over time), MEG simultaneously records thousands of tiny magnetic fields of electrical currents that occur in their dendrites in varying strengths when neurons are excited. In this case, we therefore also speak of the MEG as an oscillatory current-time diagram. Both methods are primarily used to find out how strongly a specific pain event excites the entire brain and at what frequencies and with what intensity these oscillations manifest themselves over time. These oscillations are characterized by a broad frequency range, spanning from below 0.1 to more than 100 Hz, and tend to occur in specific frequency bands that are unique to different brain structures and states (Singer, 2018). A classic functional classification of brain electrical frequency bands differentiates between delta (0.1–4 Hz), theta (4–8 Hz), alpha (8–13 Hz), beta (13–30 Hz), and gamma (30–100 Hz) bands.

Studies examining the central processing of pain in healthy (Bromm et al., 1984; Gross et al., 2007; Zhang et al., 2012) and chronic pain patients (Treede, 2003; Truini et al., 2003) have predominantly utilized phasic pain stimuli lasting from milliseconds to seconds. Consequently, these findings are likely indicative of the processing of acute pain. Phasic painful stimuli typically elicit transient spectro-temporal modulations that manifest as either an increase (event-related synchronization, ERS) or a decrease (event-related desynchronization, ERD) in oscillatory power. These transient modulations of pain-related cortical oscillatory activities are typically confined to specific frequency bands (Ploner et al., 2017): (1) an increase in the theta range (4–7 Hz) occurring between 150 ms and 400 ms after stimulus presentation and likely originating from multiple areas such as the sensorimotor cortex, anterior/mid-cingulate cortex, insula, and the secondary somatosensory cortex. These responses correspond with the extensively studied pain-related brain potentials. (2) Also, phasic pain stimuli transiently suppress alpha (8–13 Hz, α-ERD) and beta (14–29 Hz, β-ERD) oscillations in occipital and sensorimotor areas between 300 to 1,000 ms post-stimulus and (3) enhance gamma (30–100 Hz, γ-ERS) oscillation over the contralateral sensorimotor cortex. The functional meaning of these components within pain processing is still elusive and not fully understood. For example, bottom-up variations of stimulus intensity (Gross et al., 2007; Hauck et al., 2015; Tiemann et al., 2015) and top-down variations of attention (Hauck et al., 2007; Tiemann et al., 2010) influence all above-mentioned frequency components. In contrast, during repetitive painful stimulation, gamma oscillations better predict subjective pain intensity (Zhang et al., 2012) than any other frequency component, while during placebo analgesia evoked potentials (theta responses) are more closely related to pain than gamma oscillations (Tiemann et al., 2015). Taken together, the available evidence indicates that there is no one-to-one correspondence between pain-related brain oscillations and pain and its relation may change depending on the context. Hence, variations in pain processing within different contexts may be accomplished by the flexible routing of the information flow between brain areas (Ploner et al., 2017).

### 2.2 Brain oscillations and the perception of pain in hypnosis

The first EEG studies on hypnosis between 1940 and about 1980 aimed to determine whether the brain electrical activity during the hypnotic state corresponds to the EEG of sleep stages or the EEG of the waking state. Another central question was whether a characteristic EEG activity can be assigned to the degree of hypnotizability. However, the studies presented to date on the characterization of both questions revealed very contradictory and inconsistent data. On the one hand, clear synchronization patterns were found in the alpha band and other very slow frequency bands, while in other studies the exact opposite, increased oscillations of waking consciousness or even faster oscillations were found (for an extended review of these studies see Larbig and Miltner, 1993). This heterogeneity of the available empirical data is based on considerable methodological differences in the individual experimental set-ups, which were generally not designed to be replicated, i.e., they demonstrate a lack of detailed information on the induction method and measures for the control groups, on the electrode placements or information on whether the EEG recordings were carried out with the eyes open or closed and the methods used for the frequency analyses. Often no control groups were used in the experimental designs, the samples were usually too small, the EEG baseline recordings were too short, and intermittent sleep behavior was not controlled continuously or at all and documented accordingly. It also became clear that it is not possible to speak of “the hypnosis”, but that very different states can be subsumed under this term. It can therefore not be expected to identify one single replicable and always present specific central nervous correlate of hypnosis at the global level of the EEG or MEG, i.e., in a specific frequency band (for a full review of these studies, see Larbig and Miltner, 1990). Because of this, it is not possible to find a single, consistent, and always-present unique central nervous system correlate of hypnosis at the global level of the EEG or MEG, that is, in a certain frequency band.

In addition to this contradictory state of affairs, it should be noted that to date there are only a small number of experimental studies that have examined the effects of hypnosis on pain processing in conjunction with possible changes in brain electrical frequency bands (Croft et al., 2002; De Pascalis et al., 2004; Houzé et al., 2021). Croft et al. (2002) investigated the relation between pain-related cortical oscillations (alpha, beta, gamma) and the subjective pain intensity in response to phasic painful stimuli in 33 subjects (16 lows, 17 highs) during three conditions: control, hypnosis, and hypnosis with hypnotic analgesia suggestions. In the control condition, only prefrontal gamma activity (32–100 Hz) predicted subjective pain ratings both in low and high hypnotizable subjects. This relation remained unchanged in lows during hypnosis but was no longer evident in highs indicating that hypnosis differentially alters the pain-gamma relationship.

In a similar study, De Pascalis et al. (2004) investigated the phase reordering of gamma activity during waking (control), hypnosis, and posthypnotic condition. During these conditions, subjects (12 low, 13 medium, and 13 high hypnotizable subjects) additionally received a suggestion of focused analgesia aimed at reducing the perception of painful stimuli by producing an obstructive hallucination. In each condition, subjects completed an oddball paradigm composed of infrequent and frequent painful electrical stimuli that were delivered at a constant inter-stimulus interval. Correlational analysis of EEG trials from each participant unveiled short periods of gamma pattern phase ordering, occurring before and after the presentation of a stimulus. These intervals persisted for approximately six cycles. High and low hypnotizable subjects showed significantly reduced phase-ordered gamma scores during hypnotic and post-hypnotic analgesia compared to waking without analgesia and hypnosis without analgesia. Across the waking conditions (painful stimulation with and without focused analgesia), phase-ordered gamma scores at central electrode sites predicted subjective pain ratings similarly in low, medium, and high hypnotizables, while there was no significant relationship during hypnotic conditions for high and medium hypnotizables. These results suggest that hypnosis affects the neuronal timing of gamma oscillations, which may lead to changes in pain perception.

Finally, a study by Houzé et al. (2021) investigated the efficacy of suggestions given with and without hypnotic induction and compared hypnotic analgesia to a distraction condition. Noxious electrical stimulation was applied to the sural nerve and brain electrical recordings were analyzed in theta-band (0–500 ms, 3–7 Hz), reflecting the time domain related somatosensory potential, and for alpha-band desynchronization (ERD, 350–1,000 ms, 8–12 Hz). At the behavioral level, subjective pain intensity decreased in distraction, suggested-hypoalgesia, and hypnotic-hypoalgesia, but increased during hypnotic-hyperalgesia condition. The study found no differences in theta and alpha band between hypnotic hypoalgesia and hypnotic hyperalgesia. In contrast, distraction led to a significant reduction of theta power compared to hypnotic-analgesia, suggesting that the time domain related SEP components were influenced by diverting attention from painful stimuli.

In the past, brain oscillations were intensively studied in a small number of field experiments that tested prolonged painful events that more corresponded to clinical pain during autohypnotic pain rituals in different cultures, in a fakir and control subjects, and in athletes during a marathon run using telemetric EEG recordings were taken (Larbig, 1982, 1994; Larbig et al., 1982; Larbig and Miltner, 1993).

In a first study, firewalkers practicing pyrovasia in northern Greece were investigated while dancing on a fire with glowing charcoal (200–400°C) in seductive trance to block the agony of touching their bare feet (Larbig, 2015). Firewalking left no foot burns despite intense ember contact. Ancient pyromania is practiced in south-eastern Europe, Asia, India, Indonesia, and the South Sea Islands. Greeks perform firewalking for 3 days on May 21, St. Constantine and St. Helena's feast day, as an inviolability ceremony. Classical legend anchors this religious rite with Constantine's heroic act of rescuing holy Christian treasures from a burning church during Roman reign. Pyrovasia is utilized to identify with saints to learn and enforce stress- and pain-management. Preparing this dance includes several days of singing religious songs, dancing, sacrificing animals, processions with symbols, and contemplative exercises. Multiple hand touches on the candle flame check the firewalk's trance depth before walking on the charcoals. The firewalk EEG demonstrated clear increases in high-amplitude theta oscillations over the sensorimotor brain in all direct foot-ember interactions. Multiple repetitions of firewalking (without pyrovasie rites) in Germany with volunteers showed no pain with sole contact times below 400 ms and surface temperatures around 400°C. Some volunteers also had a slow theta EEG (Larbig and Miltner, 1993).

Another brain-electric field study of Larbig (1994, 2015) on hook swingers in Sri Lanka monitored celebrants' brain waves telemetrically throughout this centuries-old fertility ritual in honor of Mhatoba in Indian and Sri Lankan by Hindus following protracted droughts. Ropes suspend the celebrant from a wooden wagon on approx. He blesses newborn children, fruit, and grain as he is pulled over the fields with 6–8 metal hooks through the skin and muscles of the back. Celebrants stay in a trance for hours without pain. Wounds heal in a few days after wood ash is sprinkled following hook removal. Telemetric recordings of 9 hook-swinging celebrants before, during, and after the pain ceremony showed significant theta-frequency increases in anticipation and during pain stimulation over sensorimotor brain areas, which were still detectable in 5 people a few minutes after the ceremony.

In a further study on self-induced trance, a fakir was observed (Larbig, 1994, 2015) who thrust ~50 cm long dagger- and floret-like skewers through his throat, mouth, and abdomen skin demonstrated significant theta frequency increases in anticipation of and during painful brain stimulation. These findings prompted a controlled laboratory trial with the fakir and 14 controls. The hypothesis was to see if this fakir has higher high-amplitude theta activity than the control participants during anticipated painful stimulation or self-application of pain while applying autosuggestive analgesic suggestions to reduce pain. The fakir's EEG power spectrum was very different from normal participants. Slow oscillations and strong EEG synchronization occurred in anticipation and trance-like consciousness during painful stimulation. Averaged across people, experimental settings, and recordings, slow cortical potentials exhibited a negative pain anticipation shift.

To summarize these studies, the field and laboratory tests revealed that painful stimulation during autohypnotic induced trance conditions strongly slowed the spontaneous oscillations of the EEG over pain processing brain regions. These findings are consistent with numerous hypnosis experiments that showed clear synchronization patterns up to the slow sensory and emotional brain areas during the trance, while other studies reported EEG desynchronization.

In summary, current evidence underscores that different psychological modulations of pain may differentially change pain-related brain oscillations and thereby presumably enable flexible routing of information flow between brain areas.

### 2.3 ERP activities of pain processing during hypnosis and hypnotic analgesia

The next level of neurophysiological correlates of pain control includes so-called evoked (EPs) or event-related potentials (ERPs, Luck, 2014) or event-related magnetic fields (MEFs Bromm and Scharein, 1996) represent a subgroup of brain electrical parameters of the EEG. They provide temporally structured information about stimulus-dependent changes in the oscillation process of the EEG/MEG and represent brain-electric correlates of various processes, stages, and phases of the processing of stimuli, emotions, and internal cognitive processes (expectations, evaluations of stimuli, stimulus-dependent attention activities, planning, discrimination, cognitive operations, etc.) with individual potentials recognizable as amplitudes in the voltage-time diagram. In addition, there are several mathematical models and software programs that use the head distribution of individual amplitudes to analyze models of which brain regions could be constitutive as neuronal sources for the expression of individual potentials.

As many studies have shown, pain stimuli evoke a very characteristic positive and negative electrical voltage-time diagram (EEG) composed of a mixture of voltage oscillations of different frequencies and potential components (half-waves or amplitudes) of the sERP, which are contained in the EEG and can be crystallized from the EEG by averaging the voltage-time diagrams in response to a series of stimuli (Bromm and Scharein, 1983, 1990; Bromm et al., 1985; Miltner and Weiss, 1998). The composition of the EEG of different frequency bands can be determined by frequency-analytical mathematical models, either over fixed time intervals using Fourier analysis or time-synchronously by methods such as wavelet analysis (Salansky et al., 1995; Kelly et al., 1997; Wacker and Witte, 2013) and others. Depending on the time elapsed since stimulus application (latency), and the electrical orientation of the voltage-time diagram (P: positive, N: negative), in addition to the early and mid-latency components of the sERP (Schwender et al., 1997), so-called late components and ultra-late components have become especially interesting for studies on the effects of anesthetics and analgesic agents (for more details see below). The important amplitudes of such late components comprise the N1 or N100, P2 or P200, and as a combination and average of the N1 and P2 amplitudes the vertex complex, and the P300 family, of which one amplitude, i.e., the P3b proved particularly interesting. The number indicates in which order or at which time lack after stimulus onset the respective amplitude reaches its maximum (Luck, 2014). While the N1 is mainly determined by early attentional processes to physical properties like dynamics, size, etc. of the stimulus, the P2 mainly reflects the sensory quality (intensity, somatosensory aspects of stimuli like pressing, stinging, burning, etc. of the stimuli and so on Luck and Kappenman, 2011). Amplitudes of P2 and the vertex potential vary positively with physical and even more systematically with the subjectively experienced intensity of a stimulus (Harkins and Chapman, 1978; Bromm, 1991; Miltner and Weiss, 1998). That is, the greater the physical and subjectively felt intensity of the applied stimulus, the greater the amplitude of these potentials. The subjective meaning of the stimulus (for example the degree of its danger, threat, and riskiness) and its importance to the individual as well as its task relevancy manifests itself somewhat later in the P3b and the succeeding slow wave (not further treated in this review, for a comprehensive summary of the P300 family see Johnson, 1986). Ultra-late components have been shown to emerge much later around 1,200–1,500 ms post-stimulus and mainly reflect the neural activities that emerge from slow-conducting unmyelinated C-fibers (Bromm, 1991; Opsommer et al., 2001). Because of their close relationship to the intensity of pain stimuli, the vertex potential, the P2 and the P3b have been described as “quasi-objective measures” of pain (Bromm, 1995), and have therefore often been used to assess the effect of analgesics (Chapman et al., 1990; for a summary see Bromm and Scharein, 1990) and psychological pain treatments, i.e., distraction (Miltner et al., 1989), mindfulness (Cahn et al., 2013; Jo et al., 2016; Aly et al., 2023), and hypnosis (see below).

In the early 1960s, Halliday and Mason (1964) examined how hypnotic anesthesia affects cerebral evoked responses to electrical, mechanical, and auditory stimuli. Nine healthy volunteers were given electrical or mechanical index finger stimulation or auditory stimuli and suggested that the stimulated would become numb and frozen, and any feeling of the hand would be lost or suggested hypnotic deafness during presentation of auditory stimuli. The stimuli did not produce the expected effects. The sERP and aEPRs did not change when individuals viewed the stimuli as instructed. Even with distant or barely audible “clicks” during auditory stimulation, aERPs remained like the control condition.

Amadeo and Yanovski (1975) conducted a study on the relationship between hypnotic and non-hypnotic states, attention, and event-related responses (ERPs) following random painful stimuli. The study involved five participants with previous experience in hypnosis experiments. Somatosensory brain electrical responses (sERPs) and auditory brain electrical responses (aERPs) were measured using strong electrical pulses and clicks, respectively. The study compared non-hypnotic and hypnotic states and found that late ERP responses were increased in the non-hypnotic state, while the hypnotic state showed no changes compared to the resting condition. Surprisingly, ERP amplitudes were greater when subjects responded to clicks than to shocks.

In a study conducted by Arendt-Nielsen et al. (1990), eight highly hypnotically sensitive adults participated. The study aimed to investigate pain-associated brain measures following suggested pain analgesia. The participants underwent a hypnotic induction to achieve a deep trance state and were given suggestions for hyperalgesia (increased pain sensitivity) and analgesia (decreased pain sensitivity). During the hyperalgesic phase, participants were told that their right hand was immersed in hot water (Cold Pressure Test CPT, Hines and Brown, 1933; Velasco et al., 1997) and would feel unpleasant or painful. Evoked potentials were recorded using a needle electrode inserted into the vertex skin and referenced to the earlobes. The results showed that pain thresholds increased during hypnotic analgesia and decreased during hyperalgesia compared to a baseline without suggestions. The vertex component of the brain displayed greater amplitude potentials during hyperalgesia and lower magnitudes during hypnotic analgesia compared to the waking baseline.

Zachariae et al. (1991) investigated 12 highly sensitive participants in seven experimental conditions: baseline, neutral, anger, fear, sadness, happy, and posthypnotic awake control using brief laser stimuli on the dorsal part of the hand. The stimulus intensity was 1.5 times the individual's predetermined pain threshold. As in the former study, ERPs were also recorded using a needle electrode from the vertex to connected earlobes for each condition. Participants hypnotically recalled a former emotional occurrence under emotional circumstances to elicit the four emotional states. The neutral hypnotic control condition did not imply pain. Participants were told to relax and forget memories and emotions between emotional states. A visual analog scale was used to measure laser stimulation pain and emotion retrospectively. After each emotional condition, anger recall was 79.6%, fear 87.5%, depression 63.8%, and happiness 82.1% compared to pretraining. sERPs indicated reduced pain electrical activity in angry situations and enhanced activity in depressive situations compared to baseline. EEG power was strongly associated with depression severity but not the other three emotions.

Miltner et al. (1993) conducted a study to evaluate the processing of painful vs. nonpainful electrical stimulation and visual stimuli in 16 healthy participants. The participants were exposed to two hypnotic suggestion conditions, including a hypoalgesic condition where they were suggested to wear an analgesic glove and a hyperalgesic condition where pain was suggested to be enforced. The intensity of the stimulation applied during these conditions was predetermined for each participant. The researchers recorded pain ratings, sERP responses, and baseline ERPs. They also monitored EOG, ECG, and skin temperature. The results showed that treatment significantly affected pain ratings, with anesthetic pain being half as intense as hyperalgesic pain. There were no significant changes in sERP amplitudes for the P260 and P300 and also not for the N150 compared to baseline. The VEP did not show any significant differences.

These results were replicated by Meier et al. (1993) conducted a study to investigate the effects of hypnotic analgesia and hyperalgesia on the subjective evaluation and processing of intracutaneous electric stimulation to a finger. The study included 9 participants with no previous experience with hypnosis. Besides the intensity of stimuli, brain measures, including late sERPs, auditory aERPs, and power spectral density (PSD) of EEG segments, were recorded and analyzed. Hypnosis significantly alleviated pain in the analgesia condition and increased pain in the hyperalgesia condition. The hypnotic suggestion had no effect on sERP and aERP amplitudes. The PSDs calculated from spontaneous EEG before each stimulation were not influenced by hypnosis.

In the following study by Zachariae and Bjerring (1994) pain-evoked potentials were measured in response to laser pulses in participants who underwent a neutral hypnosis, hypnotic analgesia with relaxation, distracting imagery, sensory pictures of anesthesia, and numbness, as well as a placebo condition. The study also assessed cognitive differences in hypnotic susceptibility using a questionnaire on mental imagery vividness. The participants were divided into highly and low hypnotizable groups based on their hypnotic susceptibility scores. Pain thresholds were measured before hypnosis, and evoked potentials were measured in response to laser stimulation. EEG recordings were taken at the vertex as the maximum vertical. Sensory pain ratings were obtained after laser stimulation using visual analog scales. The results showed that all treatments, except placebo, reduced pain in both high- and low-hypnotizable participants. However, high hypnotizable participants showed greater reductions in pain compared to low hypnotizable participants. When comparing evoked potential amplitudes to the baseline, only the high-hypnotizable group had significant reductions.

Crawford et al. (1998) conducted a study on hypnotic analgesia in 15 participants with chronic low back pain who were able to significantly reduce pain perception following hypnotic analgesia instructions while exposed to the CPT. The study consisted of three sections, including pain history interviews, mental examinations, and a CPT to assess pain control. Participants received hypnotic analgesia training. In Session 2, participants listened to hypnotic analgesia procedures and assessed somatosensory pain-related stimuli during wakefulness and hypnosis. Pain ratings were measured on a scale of 0 to 10, with higher ratings indicating more pain, and magnitudes and latencies of artifact-free P70, N140, P200, N250, and P300 amplitudes in anterior frontal (Fp1, Fp2), mid frontal (F3, Fz, F4), central (C3, Cz, C4), and parietal (P3, Pz, P4) regions were extracted during wakefulness and hypnotic analgesia. The study found significantly decreased pain ratings when participants were exposed to hypnotic analgesia. Hypnotic analgesia enhanced the N140 amplitude in the anterior frontal region. During hypnotic analgesia decreased spatiotemporal perception was evidenced by reduced amplitudes of P200 (bilateral mid-frontal and central, and left parietal) and P300 (right mid frontal and central). Hypnotic analgesia further led to highly significant mean reductions in perceived sensory pain and distress.

Danziger et al. (1998) conducted a study with 18 hypnotic novices who were stimulated with nociceptive electrical stimulation on their sural nerve under three conditions: control, hypnotically suggested left lower limb analgesia, and post-hypnosis. Hypnotic analgesia suggestion induced a significant elevation in pain threshold and all participants exhibited significant alterations of the RIII reflex amplitudes of 20% or more, in contrast to the control condition. The pain threshold also increased to a similar degree. Two persons showed unclear patterns of the RIII reflex during hypnotic analgesia, 11 subjects a significant inhibition of the reflex. In another group of seven subjects, a significant facilitation of the reflex was observed. Both subgroups exhibited comparable reductions in the magnitude of late somatosensory evoked brain potentials during hypnotic analgesia but no changes in the autonomic parameters or the spontaneous EEG were seen. These findings indicate that many methods of adjustment may be in action during hypnotic pain relief and that methods vary depending on the individual.

Schuler et al. (1996) conducted a study on the effects of distraction and hypnotic analgesia on pain perception and brain measures in healthy volunteers. 13 Subjects were selected for participation who could control CPT-induced pain with hypnotic analgesia. Pain intensity and aversiveness were assessed with visual rating scales. Besides the measurement of subjective pain responses to intracutaneous painful stimulation, the study also measured sERP amplitudes and EOG recordings. The results showed that distraction and hypnotic analgesia significantly reduced pain severity and aversiveness. The sERP analysis reduced the P80, P260, and P300, amplitudes in response to distraction, while hypnotic analgesia affected only the P80 component.

The study by Friederich et al. (2001) was conducted to investigate the effects of hypnosis and distraction on pain perception in a group of 16 highly hypnotizable participants. In one condition, the volunteers were hypnotized and asked to imagine wearing a glove soaked in an analgesic substance, while in another condition, they were asked to listen attentively to a tape of a short mystery novel, and in a third condition they had no further tasks and suggestions while stimulated with laser heat stimulation applied to the back of the left hand. Laser-induced LEPs were recorded from 62 EEG electrodes, and ratings of pain intensity and aversiveness were collected after each block of 10 stimuli. Pain reports were significantly reduced during hypnotic analgesia and distraction as compared to the control condition. The amplitudes of the LEP components N200 and P320 were also significantly smaller during distraction than during control. However, no significant difference in these amplitudes was obtained for hypnotic analgesia as compared to the control condition. Results indicate that hypnotic analgesia and distraction of attention obviously represent different mechanisms of pain control and might involve different brain mechanisms.

The study by De Pascalis et al. (2001) examined the electrocortical and autonomic responses to painful stimuli in 29 female undergraduates. The participants were selected based on high hypnotic susceptibility scores and were tested under different conditions including awake, relaxation with analgesia, dissociated imagery, focused analgesia, and placebo. The study measured sensory and pain thresholds using electrical stimulation and recorded EEG activity from eight electrodes. Skin conductance response (SCR) and heart rate (HR) were also measured. The results showed that deep relaxation, dissociated imagery, and focused analgesia significantly decreased pain and distress in all participants. High-hypnotizable subjects experienced greater pain reduction during focused analgesia and dissociated imagery. The N200 amplitude was higher in temporal scalp locations for moderate and low hypnotizable subjects, while highly hypnotizable subjects had larger N200 amplitudes in general. Focused analgesia enhanced the temporal N200 peak in highly hypnotizable individuals. P300 amplitudes decreased during deep relaxation, dissociated imagery, and focused analgesia in hypnotizable individuals. Skin conductance and heart rate decreased during hypnosis for all participants.

Tables 1, 2 summarizes the characteristics and effects, respectively, of the EEG studies on subjective pain perception and ERPs after noxious and non-noxious stimulation. It should be noted that the biopsychological investigation of pain processing in hypnosis research is already somewhat outdated, and peer-reviewed articles of more recent date can hardly be found in the international literature. While the first two studies by Halliday and Mason (1964) and Amadeo and Yanovski (1975) were still rather skeptical that analgesic suggestions under hypnosis have a pain-relieving effect, all nine subsequent studies that have investigated the effect of hypoalgesic suggestions agree that they have a pain-relieving effect and three out of four studies investigated hyperalgesic suggestions observed a pain-intensifying effect. However, the observations of the brain electrical amplitudes N100, P200, the vertex complex, and P300 are less clear. About the N100 and P200, five out of 8 studies agree that these components are not affected by hypnosis or hypnotic analgesia compared to a neutral control condition. But two studies found an increase and one study a decrease in the N100. Two other studies also found a decrease in the P200. The studies of our research group (Miltner et al., 1993; Schuler et al., 1996; Friederich et al., 2001) also showed that distraction has a positive, reducing effect on the P200 amplitude. Concerning the vertex complex, 3 out of 7 studies under hypnosis and hypoalgesia suggestions showed no change in amplitude strength and 4 showed a reduction in amplitude compared to the control condition. Under hyperalgesia suggestions, 3 out of 4 achieved no change and one reported an increase in amplitude. The results for the P300 showed no effect in 3 of the 5 studies testing this component, and a reduction in amplitude in two studies.

**Table 1:** Characteristics of EEG/ERP-studies on pain processing during hypnosis and hypnotic analgesia.

**Reference**	**Neuronal parameter**	**Number of subjects**	**Susceptibility test**	**Pain stimulation**	**Design conditions**	**Hypnotic suggestions/ conditions**	**Random order of conditions**	**Tasks during condition**	**Pain assessment**	**Statistical testing**
Halliday and Mason (1964)	ERP	*n* = 5	No	Electrical, non-painful stimuli (*n* = 66 or 132) at fingers	1. CON^a^ 2. HYP^b^	A) progressive relaxation B) anesthesia	No	n/a^c^	No systematic query about intensity of perception	No, descriptive
Amadeo and Yanovski (1975)	ERP	*n* = 5	No	Electrical, non-painful stimuli/shocks (*n* = 90) at the wrist, in pseudo random order with clicks	1. CON 2. HYP 3. CON	A) remember and apply former self-hypnosis experience B) - inaudibility to clicks - insensitivity to shocks - increased loudness to clicks - hyperaesthesia to shocks	No	A) remain alert B) respond to clicks C) respond to shocks	n/a	Parametric
Arendt-Nielsen et al. (1990)	ERP	*n* = 8 highs	HGSHS >9	Laser, painful stimuli at the dorsum of the hand	1. CON 2. HYP	A) standardized induction B) - hyperaesthesia - analgesia	No	n/a	Pain thresholds determined before/during hypnosis	Non-parametric
Zachariae et al. (1991)	ERP	*n* = 12 highs	HGSHS >9	Laser, painful stimuli (*n* = 16) at the dorsum of the hand	1. CON 2. HYP 3. CON	A) standardized induction Hypnotically recalled situation of: B) - anger, fear, and depression in pseudorandomized order - happy	No, Only partly hypnotic conditions	n/a	Interview after experiment (VAS^d^)	Non-parametric, parametric
Miltner et al. (1993)	ERP	*n* = 16	No	Electrical, painful stimuli (*n* = 60) at the finger	1. CON 2. HYP	A) standardized induction B) - hypoalgesia - hyperalgesia	Yes	Intensity rating	After each stimulus (VAS)	Parametric
Meier et al. (1993)	ERP	*n* = 10 highs	SHSS>3	Electrical, two painful stimulus intensities (*n* = 80) at the finger	Day0: habituation Day1: 1. CON 2. HYP Day2: 1. CON 2. HYP	A) standardized induction (SHSS) B) - hypoalgesia - hyperalgesia Order of hypnotic suggestions balanced between sessions and days	No, but order of hypnotic conditions	Intensity rating	After each stimulus (analog scale)	Parametric
Zachariae and Bjerring (1994)	ERP	*n* = 20 (10 lows, 10 highs)	HGSHS < 5 HGSHS>9	Laser, painful stimuli (*n* = 16) at the dorsum of the hand	1. Placebo (anesthetic spray) 2. HYP	A) standardized induction B) - neutral hypnosis - deep relaxation - dissociative imagery - analgesia	Yes	n/a	After each condition (VAS)	Non-parametric
Schuler et al. (1996)	ERP	*n* = 13	HGSHS	Electrical, painful stimuli (*n* = 60) at the finger	1. CON 2. Distraction 3. HYP	A) standardized induction B) analgesia/relaxation	Yes	No	After each block (*n* = 20) (intensity and aversity of last stimulus, VAS)	Parametric
Crawford et al. (1998)	ERP	*n* = 17 chronic back pain patients (1 low, 6 moderate, 8 highs)	SHSS < 3 5 < SHSS < 8 SHSS>7	Electrical, painful stimuli (*n* = 30) at the finger	1. CON 2. HYP 3. CON	A) standardized induction (shortened SHSS version) B) - attend closely hand - analgesia - attend closely hand	No	A) attend stimuli B) apply analgesia techniques	After each condition (pain and distress, analog scale)	Parametric
Danziger et al. (1998)	ERP	*n* = 18 out 26 highs reporting marked hypoalgesia	SHSS>8	Electrical, painful stimuli at the leg	1. CON 2. HYP 3. CON	A) standardized induction B) analgesia	No	n/a	Pain threshold during the middle of each condition	Parametric
Friederich et al. (2001)	ERP	*n* = 20 highs	SHSS>8	Laser, painful stimuli (*n* = 70) at the dorsum of the hand	1. CON 2. HYP 3. Distraction	A) standardized induction B) analgesia	Yes	No	After each block (*n* = 10) (intensity and aversiveness, NRS^e^)	Parametric
De Pascalis et al. (2001)	ERP	*n* = 29 (10 lows, 9 medium, 10 high)	SHSS	Electric, painful stimuli (*n* = 156, oddball, standard and infrequent target) at the wrist	1. CON 2. HYP 3. Placebo (anesthetic solvent)	A) hypnotic induction B) -deep relaxation with analgesia -dissociative imagery -focused analgesia	No, but order of hypnotic conditions	respond to target stimulus with button press	After each condition (pain and distress, NRS)	Parametric
Croft et al. (2002)	Oscillations	*n* = 33 (16 lows 17 highs)	HGSHS < 5 HGSHS>7	Electrical, painful (*n* = 550 oddball, standard and infrequent target) at the finger	1. CON 2. HYP	A) standardized induction B) analgesia	Yes	Respond to target stimulus with button press	After each condition (Likert scale)	Parametric
De Pascalis et al. (2004)	Oscillations	*n* = 38 (12 lows, 13 medium, 13 highs)	SHSC	Electrical, non-painful (*n* = 70 oddball, standard and infrequent target) at the wrist	1. CON 2. HYP	A) hypnotic induction B) - analgesia (eyes closed) - analgesia (eyes open)	Yes	Count number of delivered target stimuli	After each condition (pain and distress, NRS)	Parametric
Houzé et al. (2021)	Oscillations	*n* = 20	SHSS	Electrical, painful (*n* = 40) at the leg	1. CON 2. HYP 3. Distraction	A) Standardized induction B) - hypoalgesia - hyperalgesia	Yes	No, except for distraction (mental subtraction)	After each condition (pain intensity and unpleasantness, VAS)	Parametric

^a^control condition, ^b^hypnosis condition, ^c^not available, ^d^visual analog scale, ^e^numerical rating scale.

**Table 2:** Results of EEG/ERP-studies to investigate the felt intensity and amplitudes of brain electrical event-related responses (N100, P200, vertex complex, P300) of noxious stimuli presented during hypnotic trance (HYP) and hypo- and hyperalgesic suggestions or during a distraction condition from the pain stimuli in comparison to a trance and suggestion free control condition.

	**Pain experience**				**N100**				**P200**				**Vertex complex**				**P300**			
**Studies**	**HYP**	**Hypoalgesia**	**Hyperalgesia**	**Distraction**	**HYP**	**Hypoalgesia**	**Hyperalgesia**	**Distraction**	**HYP**	**Hypoalgesia**	**Hyperalgesia**	**Distraction**	**HYP**	**Hypoalgesia**	**Hyperalgesia**	**Distraction**	**HYP**	**Hypoalgesia**	**Hyperalgesia**	**Distraction**
Halliday and Mason (1964)		n.s.																		
Amadeo and Yanovski (1975)		n.s.								n.s.										
Arendt-Nielsen et al. (1990)		S	G											S	G					
Zachariae et al. (1991)					n.s.				n.s.				n.s.				n.s.			
Miltner et al. (1993)		S	G			n.s.	n.s.			n.s.	n.s.			n.s.	n.s.			n.s.	n.s.	
Meier et al. (1993)		S	G			n.s.	n.s.			n.s.	n.s.			n.s.	n.s.			n.s.	n.s.	
Zachariae and Bjerring (1994)		S	S	S									S	S						
Schuler et al. (1996)		S		S		n.s.				n.s.		S		n.s.		S				S
Crawford et al. (1998)		S				G				S								S		
Danziger et al. (1998)														S						
Friederich et al. (2001)		S		S		n.s.				n.s.		S						n.s.		S
De Pascalis et al. (2001)		S		S		G												S		

n.s., No significant difference to the control condition; G, significantly stronger pain sensation or higher amplitudes compared to the control condition; S, significant smaller pain sensation or amplitudes compared to the control condition.

There are many reasons for this low overall agreement. But in our opinion, they are probably very strongly related to the different hypnosis inductions and hypoalgesia and hyperalgesia suggestions, as far as the differences between inductions and suggestions of the studies were comprehensible from the sparse descriptions of the methods. Other reasons could be that the context and demand requirements for the participants, which Orne emphasized very strongly many years ago (Orne, 1962), were different, and that the experimental conditions also differed significantly. It is striking that the three studies of our study group, which were carried out at long intervals and sometimes several years apart, hardly differed from each other in terms of the effects of hypnosis and suggestions, despite the completely different test subjects and different people who carried out the studies and mediated the hypnosis (from very experienced hypnotherapists to students who read the hypnosis instructions from the text and transmitted them into the experimental room via microphone). This last reason reinforces our hope that future studies might better disclose and document which methods of hypnotic induction and suggestion were used, and perhaps experimental studies will be planned and presented in which these aspects are examined in much more detail.

### 2.4 Imaging methods (PET and fMRI) investigating pain processing during hypnosis and hypnotic analgesia

The third level of brain research on pain processing relates to imaging technologies which focus on questions regarding which brain structures are significantly involved in the processing of painful states using imaging methods such as magnetic resonance imaging (MRI/fMRI) or positron emission tomography (PET). Functional MRI detects brain activity by detecting blood oxygenation changes using magnetic fields and radio waves in a non-invasive manner. Blood-oxygen-level-dependent contrast (BOLD) is the magnetic shift in oxygenated vs. deoxygenated blood used in fMRI. Higher neural activity requires more brain oxygen. This demand briefly boosts blood oxygenation by boosting blood flow to the active region. Blood oxygenation fluctuations help fMRI map brain activity. EEG and MEG monitor brain processes in milliseconds, but fMRI resolution is still significantly slower. Because cerebral activity affects blood oxygen levels (BOLD signal), fMRI monitors them. Typically, the hemodynamic response time (HRT) is 3–6 s for the BOLD signal to reflect brain activity changes. However, fMRI can scan brain activity in great detail due to its high spatial resolution. Researchers are adopting machines with greater magnetic field strengths to reduce echo times (TEs) and increase fMRI temporal resolution. Traditional 3 Tesla fMRI scanners have temporal resolutions of 2 s, whereas seven Tesla scanners can attain 100 ms.

PET uses radioactively tagged molecules to measure bodily functions. Brain activity is seen by PET scans using radioactive tracers. Radioactive decay powers PET scans. Unstable radioactive isotopes release positrons. PET scans map radioactive tracer distribution using gamma rays that result from electron-positron annihilation. PET's highest temporal resolution for brain neural activity evaluation currently is around 50 ms. Unlike fMRI, which can only detect brain activity changes in seconds, this is far quicker.

For all three levels of research, a review of experimental work will be presented that shows the processing of pain events recorded with the help of the EEG, ERP, and the magnetic equivalent of both, and with PET and MRT and how different brain regions communicate with each other based on brain electrical oscillations to ultimately constitute the sensation of pain.^1^ The most important brain regions are presented and it will be discussed which of these structures are altered by trance and hypnotic analgesic or hyperalgesic suggestions and could represent the neuronal basis of hypnotic effects.

A PET study by Crawford et al. (1993) explored the effects of hypnosis on regional cerebral blood flow (rCBF) during ischemic pain with and without suggested hypnotic analgesia in a group of 11 healthy right-handed male students with low (0–4) or high (9–12) scores on the SHSS: C. Additionally, spontaneous EEGs were taken for frequency, brain mapping, and ERP analysis. Participants were thoroughly briefed on the procedures and had a chance to familiarize themselves with the experimental environment. rCBF measurements were taken under three conditions in both states: resting with eyes closed, undergoing an ischemic procedure for up to 15 min, and the same procedure with analgesia suggestions. The order of the last two conditions was varied among subjects. During the waking state, subjects listened to information on child development, and during rest, they were asked to think about a past long exiting trip. Ischemic pain was induced in both arms using the sub-maximum effort tourniquet technique, and the same analgesia suggestions were given in both states. All instructions were delivered via a tape recording. Subjects reported their pain and distress on open-ended scales. Results on CBF indicated changes during hypnotic analgesia, particularly noting significant bilateral activation in the orbitofrontal cortex in highly hypnotizable subjects. CBF in the somatosensory cortex area decreased for these subjects but increased for those less hypnotizable. These observations align with previous research suggesting that hypnosis requires mental effort and involves attentional and disattentional allocations. The study suggests that attended pain leads to increased CBF in the somatosensory cortex, which could also be due to increased muscle contraction during the ischemic technique. Hypnotic analgesia effectively eliminates pain perception in highly hypnotizable individuals (highs), with significant CBF increases noted in the orbitofrontal and sensorimotor cortices.

In a study by Rainville et al. (1997) the role of cortical regions involved in hypnosis and their response to suggestions were investigated. Eight participants with strong hypnotic susceptibility ratings participated in the study, which included baseline, hypnosis, and hypnosis-with-suggestion of increased or decreased unpleasantness. PET scans were used to assess stimulation intensity and unpleasantness after each scan. Significant pain-related activations were observed in SI, SII, IC, and ACC during “painfully hot” versus “neutral” subtractions from alert control scans. After hypnotic induction, painful heat engaged these four cortical regions again, suggesting a minimal effect of induction on pain-related activation. However, hypnotic suggestions for increased or decreased unpleasantness affected pain and some but not all pain-related cortical areas. Comparing rCBF variations between hypnotic suggestion and control conditions showed substantial SI, ACC, and IC pain-related activations during increasing and decreased unpleasantness. No substantial pain-evoked activity was found around SII in either the increased or decreased unpleasantness conditions. The considerable difference in participants' judgments of unpleasantness during the increased and decreased unpleasantness situations shows that hypnotic suggestions selectively influence pain affect. Three pain sites engaged during hypnotic suggestion with more activation during increased unpleasantness scans than decreased unpleasantness scans. S1 pain-related rCBF was non-significantly lower in the increased unpleasantness condition than in the decreased unpleasantness condition, showing no enhanced activation in this region. Regression analyses of unpleasantness ratings and rCBF levels across all participants and all scans collected during the hypnotic suggestion condition for each pain activation location showed that only ACC activation levels encode the felt unpleasantness of these noxious stimuli. The modulation of pain-related activity in ACC closely matches a selective shift in the perceived unpleasantness of painful stimuli. The study confirms past claims that the ACC is implicated in pain and emotions but also shows that the ACC encodes pain unpleasantness.

Another PET study by Rainville et al. (1999) investigated the effects of hypnosis and suggestions on pain perception in hypnotizable subjects using rCBF and EEG measurements to assess brain activity. The study included eight participants with moderate to high hypnotic susceptibility scores. The results showed that hypnosis alone had little effect on pain sensations, but when combined with suggestions for altered pain unpleasantness, there were significant increases in rCBF in regions of the occipital cortex and inferior frontal gyri. There were also decreases in rCBF in the parietal cortex. The study found a strong increase in rCBF in the right anterior cingulate cortex (ACC) in response to hypnosis, independent of pain. The study also evaluated the effects of suggestions alone and found widespread increases in rCBF in the frontal lobes, as well as decreases in rCBF in the uncus, orbitofrontal regions, and cerebellum. Overall, the study suggests that both hypnosis and suggestions can alter pain perception and have distinct effects on brain activity.

The research by Wik et al. (1999) comprised a sample of eight female individuals with fibromyalgia who were subjected to PET scanning during resting wakefulness and hypnotic analgesia induced through visual stimulation. The scans were standardized and evaluated to measure changes in rCBF. Post-scan pain ratings were obtained using a visual analog scale. The results showed a decrease in pain ratings and an increase in rCBF in the subcallosal cingulate gyrus, right thalamus, left inferior parietal cortex, and orbitofrontal cortex during hypnotic analgesia. In contrast, a reduction in rCBF was observed in the posterior cingulate gyrus and posterior portion of the anterior cingulate gyrus. The findings suggest that hypnotic analgesia alters the individual's state of consciousness and prioritizes external suggestions over regular observation.

The study by Faymonville et al. (2000) used PET to examine the effects of hypnosis on the brain's response to noxious stimuli. The study included 11 healthy volunteers who underwent scans in three different states: hypnotic, resting, and mental imagery. The participants were exposed to warm non-noxious and hot noxious shocks to the right thenar eminence, and their pain and discomfort levels were measured. The results showed that hypnosis reduced the intensity and unpleasantness of noxious stimuli. Increased cerebral blood flow was observed in the thalamic nuclei, anterior cingulate cortex, and insular cortices in response to noxious stimuli. During hypnosis, the anterior cingulate cortex and right extrastriate region were significantly activated. The interaction analysis revealed that hypnosis affected pain perception and unpleasantness differently compared to the control states, with specific involvement of the anterior cingulate cortex. In conclusion, hypnosis can modulate the intensity and unpleasantness of noxious stimuli, and this effect is mediated by the anterior cingulate cortex.

This work by Hofbauer et al. (2001) used positron emission tomography (PET) to indirectly assess the brain activity triggered by pain, both before and after using hypnotic suggestions to regulate the perceived intensity of painful stimuli. The strategies used in this investigation were comparable to those used in a prior study conducted by Rainville et al. (1997) which aimed to manipulate the perceived unpleasantness of painful stimuli. During the experiment, 10 participants underwent scanning while being exposed to tonic warm and noxious heat stimuli on their hand. The study consisted of four conditions: alert control, hypnosis control, hypnotic recommendations to enhance pain intensity, and hypnotic suggestions to reduce pain intensity. The study demonstrates that heat stimuli administered during both the awake and hypnosis-control conditions consistently stimulated contralateral structures, including as the main somatosensory cortex (S1), secondary somatosensory cortex (S2), anterior cingulate cortex (ACC), and insular cortex (IC). The manipulation of hypnotic techniques to alter the intensity of pain sensation resulted in notable alterations in the activity of S1, as opposed to the Rainville et al. (1997) study, where the targeted manipulation of pain unpleasantness (emotional response) caused distinct changes in the ACC, regardless of pain intensity. The observed twofold dissociation of cortical modulation suggests that the sensory and classical limbic cortical regions have distinct roles in processing the sensory and emotional aspects of pain, indicating a relative specialization.

The study by Faymonville et al. (2003) involved 19 unpaid volunteers from a pool of 50 individuals who underwent screening. The participants had a high level of hypnotizability assessed as 8 out of 12 on the SSHS-Form C. Data from PET was gathered under three conditions: a state of hypnosis, mental visualization, and a state of rest. Additionally, data were gathered during two forms of stimulation: hot noxious stimulation and warm non-noxious stimulation using a thermode to the hand. During the hypnotic condition, participants were prompted to remember pleasurable personal memories. The hypnotic state was induced by ocular fixation, a 3-min muscle relaxation procedure, and permissive and indirect instructions, customized to the subject's behavior and requirements. The existence of a hypnotic state was ascertained by the observation of rolling eyes, along with the subject's verification through a deliberate foot movement. During the mental imagery, participants were instructed to vividly imagine a positive personal memory and were cautioned about entering a hypnotic state. During the resting state, participants were directed to attain a level of calm and empty their minds. After each scan, participants rated the intensity of the painful stimulation using a standard rating scale. The individuals' perception of pain when at rest was significantly reduced during the hypnotic state, but there was no notable decrease during the mental imagery condition. Given the absence of any notable disparity in pain perception between periods of rest and mental imagery, the PET data of both conditions were merged for further analysis. Compared to states of rest and mental imagery, the hypnotic state enhanced the functional modulation of the midcingulate cortex (specifically area 24a), the bilateral anterior insular cortices, pregenual anterior cingulate cortex (Brodmann's area 32), pre-supplementary motor area (pre-SMA; area 6), right prefrontal cortex (area 8), right thalamus, right striatum, and brainstem. Additionally, lesser significance levels revealed the presence of the left prefrontal cortex (area 10), right prefrontal areas 9 and 11, and the mesiofrontal cortex (area 9). The bilateral occipital cortex was the only area that showed a decrease in its functional connections with the midcingulate cortex during hypnosis, in comparison to the state of normal awareness.

A study by Schulz-Stübner et al. (2004) investigated the neural correlates of hypnosis-induced analgesia using fMRI technology (1.5 T Philips Gyroscan Intera). BOLD signals were measured in response to thermal pain to examine changes in pain perception due to hypnosis. The study involved 12 healthy volunteers who underwent a hypnotic technique involving fixation while receiving suggestions of heavy and warm body sensations. The study design included an event-related approach, with participants randomly assigned to one of two groups. Results showed that hypnosis could be induced within 2 min in all subjects, with the hypnotic state maintained during imaging. Subjects under hypnosis reported either no pain or significantly reduced levels of pain compared to stimulation without hypnosis. Brain imaging results revealed activation of the known pain network without hypnosis, while under hypnosis, new activation was found in the anterior basal ganglia. Decreased activity and reduced regional blood flow were observed in the insula, middle cingulate gyrus, and primary sensory cortex under hypnosis. The left hemispheric anterior cingulate cortex showed increased activity, while the right hemispheric anterior cingulate cortex remained unchanged.

The study conducted by Vanhaudenhuyse et al. (2009) aimed to investigate changes in brain activation and connectivity during hypnosis compared to normal wakefulness. Thirteen healthy volunteers were recruited, and a thulium-YAG laser fMRI paradigm was used. The hypnotic state was induced through muscle relaxation, eye fixation, and recollection of positive memories. Laser stimuli were administered to the left hand, and participants rated their sensory perception. The study found no significant differences in laser intensities between wakefulness and hypnosis. Non-painful stimuli activated the primary somatosensory cortex, insula, and brainstem, while painful stimuli activated additional regions such as the thalamus and anterior cingulate cortex during wakefulness. However, during hypnosis, there was reduced activation in these regions in response to sensory inputs of the same intensity. The study also found increased connectivity between the primary somatosensory cortex and remote cortices during hypnosis. The results suggest that hypnosis can modulate the neural processing of sensory stimuli in the brain.

The primary objective of the next investigation by Nusbaum et al. (2011) used PET imaging to investigate the brain networks involved in hypoalgesia, or reduced pain sensitivity. The study included 14 male participants with chronic low-back pain. The participants underwent PET scans under two conditions: normal alertness and hypnosis. They were divided into two groups, with one group receiving direct suggestions addressing pain and the other group receiving indirect suggestions focusing on overall wellbeing. The participants rated their pain levels using a VAS before and after each condition. The PET scans revealed that analgesic suggestions, whether administered during normal alertness or hypnosis, activated shared brain areas, including the medial prefrontal cortex, superior temporal gyrus, and anterior insula. Deactivations were observed in the cuneus, parahippocampal gyrus, and middle temporal gyrus. Comparing the normal alertness state to rest, analgesic suggestions activated the superior temporal and orbitofrontal gyri, inferior frontal cortex, and cerebellum, while deactivations were seen in the middle occipital and somatosensory cortices, precentral gyrus, and inferior parietal lobule. Comparing the hypnotic state to rest, analgesic suggestions activated the anterior insula, nucleus accumbens, lenticular and caudate nuclei, and anterior cingulate cortex, while deactivations were observed in the precuneus and posterior cingulate cortex. Overall, the study demonstrated that analgesic suggestions reduced pain sensation, with a greater effect observed during hypnosis. The findings support the efficacy of hypnosis in modulating pain perception and highlight the involvement of cognitive-sensory and emotional-weighted brain networks.

The study by Casiglia et al. (2020) examined the functional differences in brain areas activated during painful stimuli before and after hypnotic suggestion of hypnotic analgesia using fMRI. The study included 20 highly hypnotizable volunteers, approximately 30 years of age. The participants underwent hypnotic induction and were verified to be in a hypnotic state by observation of behavioral responses. The fMRI scans were performed on a 1.5 Tesla Philips Achieva system, and participants were instructed to immerse their left hand in icy water or rest during the scans. Pain intensity and tolerance were measured using a visual analog scale and the cold pressor test. The fMRI data showed brain activation in specific Brodmann areas during pain without analgesia and a different activation pattern during hypnotic analgesia. Specifically, BA 9, 32, 25, and 47, as well as the caudate cortex and cerebellum, were activated during hypnotic analgesia, while BA areas 1, 2, and 3 were deactivated. These findings suggest that hypnotic analgesia blocks the transmission of pain signals to primary sensory areas rather than simply dissociating the experience of pain.

The objective of the study by Desmarteaux et al. (2021) aimed to investigate how verbal hypnotic suggestions can affect perceptual processes during hypnosis. Brain activity was measured using BOLD-fMRI in a sample of 24 healthy individuals. Participants were exposed to verbal suggestions to induce hyperalgesia, hypoalgesia, or a normal sensation. A sequence of aversive electrical shocks was administered to evaluate neural responses associated with pain. The brain responses to the suggestions were used to predict changes in pain-related responses using delayed regression analysis. The study found that verbal suggestions influenced the perception of pain, as indicated by pain reports. Brain imaging analysis revealed correlations between brain activity associated with suggestions and changes in brain responses to shock in specific regions involved in pain processing. The study also identified distinct sub-regions within the pain network involved in hyperalgesic and hypoalgesic effects. The parahippocampal complex was found to play a role in contextualizing and modulating pain perception. Overall, the study highlighted the potential of verbal suggestions in modifying subjective experiences during hypnosis.

Tables 3, 4 summarizes the characteristics and effects, respectively, of the imaging studies on pain processing during hypnosis and hypnotic analgesia/hyperalgesia. When we summarize these PET and fMRI studies, we are noticeably surprised that these imaging devices, some of which differ considerably in function and technology, differ so little from each other in some identified brain regions and have produced significantly more coherent results than the studies on event-related potentials. More important for the present study, however, is the fact that the use of these methods to test activated responses of the brain under hypnosis and hypnosis-suggested analgesia and hyperalgesia yielded such a high degree of agreement with existing PET and fMRI studies of regions activated during pain. They demonstrate that hypnosis and the suggestion of hypnotically induced hypo- and hyperalgesia affect many of the brain regions indicated for pain processing and thus lay the neuronal foundation for the effect of hypnotic interventions on pain. If we consider the brain regions outlined above, which underlie the experience of pain as a unit of basic cognitive, emotional, and evaluative functions, hypnosis and its suggestions modify the neuronal activity of all brain structures that are central to the emotional aspects of pain, its sensory aspects, and the assessment of its intensity. In a majority of the studies, for example, corresponding activations/deactivations in the primary and secondary somatosensory brain areas and in downstream structures of area S1 a+b, which are relevant for the body localization of the painful event and the recognition of the sensory stimulus quality, e.g., as burning, pressing, pulling, stinging and its dynamic properties (fast, deep, broad, etc.), are found by the inhibitory or reinforcing quality of the suggestion. There is also activation/deactivation of parts of the insula that are responsible for reading and transmitting information from areas of the brain stem about heartbeat, blood pressure, and other body regulatory processes to the conscious mind. The anterior cingulate cortex is activated in interaction with its middle and posterior regions in response to the pain stimulus as horrible, disgusting, paralyzing, terrifying, etc., and attention is organized toward or away from the pain stimulus in frontal brain regions and parietal areas, e.g., the precuneus. Many other regions join in and make it clear that the hypnotic state and suggestions not only modify the conscious experience accessible to us, but also its basic neuronal functions. Everything we experience as hypnosis is not imaginary, but closely linked to neuronal processes in our brain.

**Table 3:** Characteristics of fMRI/PET-studies on pain processing during hypnosis and hypnotic analgesia.

**Reference**	**Method**	**Number of subjects**	**Susceptibility test**	**Pain stimulation**	**Design conditions**	**Hypnotic suggestions/ conditions**	**Random order of conditions**	**Tasks during condition**	**Pain assessment**	**Statistical testing**
Crawford et al. (1993)	PET	n=11 (6 lows, 5 highs)	HGSHS < 5 HGSHS>7	Ischemic pain	1. CON^a^ 2. HYP^b^	A) hypnotic induction B) analgesia	Yes	n/a^c^	Pain and distress (open-end NRS^d^)	Parametric
Rainville et al. (1997)	PET	n=5 (moderate to high)	SHSS	Immersing hand in warm(35°C) or hot water (47°C)	1. CON 2. HYP	A) hypnotic induction B) -increased unpleasantness -decreased unpleasantness	No	n/a	Intensity and unpleasantness (NRS)	Parametric
Rainville et al. (1999)	PET	n=8 highs	SHSS>8	Immersing hand in warm (35°C) or hot water (47°C)	1. CON 2. HYP	A) standardized induction B) -increased unpleasantness -decreased unpleasantness	No, but order of hypnotic unpleasantness conditions	No	Intensity and unpleasantness after each functional scan (NRS)	Parametric
Wik et al. (1999)	PET	n=8 highs patients with myofibralgia	HGSHS>9	No, chronic pain	1. CON 2. HYP	A) hypnotic induction B) analgesia	Yes	Watch videotapes	After each functional scan (VAS^e^)	Parametric
Faymonville et al. (2000)	PET	n=11 highs	SHSS>8	Thermal stimulator, warm (39°C) and hot (47°C) stimulation of the thenar of the hand	1. CON 2. HYP 3. Mental imagery	A) hypnotic induction	No, but order of CON and Mental Imagery	CON: empty mind HYP: re-experience autobiographical memory Mental imagery: imagine autobiographical memory	After each functional scan rating of intensity and unpleasantness	Parametric
Hofbauer et al. (2001)	PET	n=10 (SHSS ranging from 1–10)	SHSS	Immersing hand in warm (35°C) or hot water (46–47.5°C)	1. CON 2. HYP	A) standardized induction B) -increased intensity -decreased intensity	No	No	After each functional scan rating of intensity and unpleasantness	Parametric
Faymonville et al. (2003)	PET	n=19 highs	SHSS>8	Thermal stimulator, warm (39°C) and hot (47°C) stimulation of the thenar of the hand	1. CON 2. HYP 3. Mental imagery	A) hypnotic induction	No, but order of CON and Mental Imagery	CON: empty mind HYP: re-experience autobiographical memory Mental imagery: imagine autobiographical memory	After each functional scan rating of intensity (NRS)	Parametric
Schulz-Stübner et al. (2004)	fMRI	n=12	No	Thermal stimulator, warm (39°C) and hot (47°C) stimulation	1. CON 2. HYP	A) hypnotic induction	Yes	CON: think “something nice”	Yes, not specified	Parametric
Vanhaudenhuyse et al. (2009)	fMRI	n=13 highs	HGSHS>7	Brief non-painful and painful laser heat stimulation (n=200) of the hand	1. CON 2. HYP	A) hypnotic induction	Yes	Stimulus rating	After each stimulus rating of intensity (NRS)	Parametric
Nusbaum et al. (2011)	PET	n=14 chronic low-back patients	SHSS>3	No, chronic pain	1. CON 2. HYP	A) hypnotic induction	No	Stimulus rating	After each stimulus rating of intensity (VAS)	Parametric
Casiglia et al. (2020)	fMRI	n=20 highs	SHSS	Immersing hand in ice water (CPT^f^; maximum 120 s)	1. CON 2. HYP	A) hypnotic induction B) analgesia	Yes	Immerse/remove hand in/from ice water	Intensity after each functional scan (VAS)	Parametric
Desmarteaux et al. (2021)	fMRI	n=24	SHSS	Electrical, painful stimuli (n=18) at the leg	1. HYP	A) standardized induction B) -hypoalgesia -hyperalgesia	Yes	No	Intensity and unpleasantness after each block scan (VAS)	Parametric

^a^control condition, ^b^hypnosis condition, ^c^not available, ^d^numerical rating scale, ^e^visual analog scale, ^f^cold pressor test.

**Table 4:** Results of experimental studies on activation/deactivation of brain regions involved in the processing of experimental pain (column 2) and during suggestions of hypnosis and hypnoptic analgesia (columns 3–14).

**Major brain areas of pain processing**	**Activation and functions due to application of experimental pain reported in studies of footnote (1)**	**Crawford et al. (1993)**	**Rainville et al. (1997)**	**Wik et al. (1999)**	**Rainville et al. (1999)**	**Faymonville et al. (2000)**	**Hofbauer et al. (2001)**	**Faymonville et al. (2003)**	**Schulz-Stübner et al. (2004)**	**Vanhaudenhuyse et al. (2009)**	**Nusbaum et al. (2011)**	**Casiglia et al. (2020)**	**Desmarteaux et al. (2021)**
Brainstem	Regulation of physiological adjustment							IC		DC			
Thalamus	SS, I, E			IC				IC		DC			
Gyrus postcentralis S1	SS, I	IC	IC				IC		DC	DC		DC	DC
Gyrus postcentralis S2	SS, I, Loc		IC				IC					DC	
Gyrus postcentralis S3 a+b	SS, I											DC	
Insular cortex	SS, I, E		IC	IC			IC		DC	DC	IC		DC
Frontal cortex	Att				IC								
Prefrontal, dorsolateral, inferior frontal, ventrolateral cortex	Att				IC			IC			IC	IC	
Orbitofrontal cortex	I	IC		IC	DC			IC					
Parietal cortex/operculum	IC			IC	DC								DC
Posterior cortex	IC										DC		
Occipital cortex / Precuneus	Att				IC						DC		DC
ACC	E		IC	DC	IC	IC	IC	IC	IC	DC	DC/IC	IC	
Pre-and midcingulate cortex	IC							IC	DC				DC
Posterior cingulate gyrus	IC			DC									DC
Subcallosal cingulate gyrus	IC			IC									
Subgenual cingulate cortex	IC											IC	
Striatum	Integration of sensorimotor, emotional and motivational functions							IC					
Nucleus accumbens	Processing of divergent feelings of pain										IC		
Amygdala	IC												
Locu coeruleus	IC												
Parahippocampus	IC										DC		IC
Uncus					DC								
Cerebellum					DC							IC	
Pre-SMA BA 6								IC					DC
Primary motor area MA													DC
Basal ganglia									IC				

Cells of columns 3–14 containing letters got affected by painful stimulation while Ss were exposed either to hypnosis or hypnotic analgesic suggestions; in case of empty cells we found no classifyable information; Att, activation relevant for Attention; DC, Decreasing activity, IC, Increasing activity, I, Relevant for processing of pain intensity, Loc, Relevant for stimulus localization, SS, Relevant for somatosenzation, E, Relevant for emotional processing. (1) Compilation from following publications: Bornhovd et al. (2002),Treede (2003),Bromm (2004),Apkarian et al. (2005),Kuner (2010),Baliki and Apkarian (2015),Segerdahl et al. (2015),Bastuji et al. (2016a,b),Bliss et al. (2016),Sherman (2016),Taylor and Westlund (2017),Garcia-Larrea and Bastuji (2018),Groh et al. (2018),Tajerian et al. (2018),Corder et al. (2019), and Del Casale et al. (2022).

## 3 Discussion

All three research approaches of EEG/MEG, ERPs and the two imaging methods fMRI and PET have shown that the changes induced in experience and behavior by hypnosis are accompanied by systematic activation changes in regions of the brain that are relevant for the processing of noxious stimuli and the production of pain. Regarding the somatosensory aspects of the pain experience, such as the body localization of the noxious stimulus and its somatosensory properties as pecking, stinging, pulling, drilling or hammering, etc., or concerning its intensity, the majority of patients do not experience pain in the same way. In the majority of studies that investigated the many regions depicted in Table 2, deactivations in the primary somatosensory cortical fields and association fields as well as the insular cortex occur, which is also reflected in some ERP studies in significantly smaller brain electrical responses of the P200 and P300 amplitudes of the sERP compared to painful stimuli presented in control conditions without hypnosis and hypnotic suggestion. In contrast, S2 shows increased activation in the studies by Rainville et al. (1997) and Hofbauer et al. (2001) when strong stimuli were also perceived as particularly unpleasant. This indicates that this region responds primarily to emotional aspects of the pain stimuli in addition to its somatosensory properties. In studies in which this differentiation between strength and affect type was not primarily investigated, deactivation of the insula was observed, and in almost all studies except that of Wik et al. (1999) the affective control of stimuli under hypnosis or hypnotic analgesia was accompanied by coherent activation, indicating that this region was not particularly affected by the analgesic content of suggestions that focused on reducing stimulus strength. Based on physical reasons that the strength of any dipole diminishes by the square of the distance between source and electrode (Luck and Kappenman, 2011; Luck, 2014), this difference will hardly be reflected in the sERP amplitudes, as the generators of the brain electrical processes of the ACC as well as of the insula that a localized ~3–4 cm below the cortex surface in the middle of the brain hardly become expressed in the averages of the sERP by the relatively smaller numbers of stimuli applied in such studies. Stronger activations can also be seen in the orbitofrontal cortex and various other frontal regions. The orbitofrontal cortex is attributed with the function of being actively involved in the evaluation of emotional stimuli within the context of attended tasks, particularly when it comes to learned emotions (Rolls et al., 2020). However, a distinction must be made here between early and late attention processes, because in many of the sERP studies reported, there was hardly any deactivation of the N100 amplitudes in frontal brain regions under hypnosis or analgesic suggestions (see Table 2), which indicates that stimuli are not blanked out under hypnosis. In later amplitudes, such as the P200 and especially the P300, there were even slight increases observed under hypnosis compared to control stimuli and followed the functional. This indicates that these stimuli are particularly task significant/relevant due to the suggestions since their processing should be changed and thus expressed in sERP amplitude as increased, when a stimulus was assigned a special task (Johnson, 1986). The aim here of the mental and neural systems of the hypnotized person is not to modify all surrounding stimuli, but only the one that causes pain. Our group's previously outlined sERP studies show that acoustic stimuli applied shortly before the pain stimulus were neither masked out nor modified in their physical dimension. The brain electrical amplitudes of the N100 of all three studies by Miltner et al. (1993), Schuler et al. (1996), and Friederich et al. (2001) and the study by Meier et al. (1993) were unchanged and the whole sERP was characterized by a voltage-time diagram that was similar to those observed for these stimuli without hypnosis. Similar activation was observed for the P300 amplitude, i.e., no chance as a function of hypnosis or suggestion of analgesia.

Both tables and the accompanying text initially give the impression that neuroscientific research into hypnosis and hypnotically induced analgesia is primarily contradictory and has produced hardly any consistent results. When looking at the studies and the many differences in hypnotic induction, the verbal content of analgesic suggestions, the various experimental conditions, research and evaluation methods and strategies, and, above all, the many different questions, this is not surprising, because the neuronal response and the interactions between all parts of the brain involved in pain and hypnotic processes react to all differences in a highly sensitive way, so that the prospect that these studies will show any kind of replication beyond very large general structures would be presumptuous. Obviously, every distinguishable aspect of the stimuli to be processed changes a complex network of neuronal structures, so it would be quite unusual if studies as different as the ones described here showed close correspondence in many neuronal details. This would require much more detailed and phenomenally differentiated experimental designs and experimental conditions that would have to be tested against each other. What was important for us in this review was the fact that, at a very rough level of analysis, the various studies at different levels of neuronal methods showed on average clear differences in neuronal activation during the processing of pain under hypnosis and hypnotic analgesia compared to control conditions. The closer the experimental designs and methods matched between the studies, the more similar the global findings were. Given the very different questions, hypnosis inductions, pain stimulation models, suggestion contents and groups of people in the experimental neuroscientific studies on the effects of hypnosis and analgesic suggestion summarized above, it would be desirable for the future of this research, that these aspects would be explored much more systematically and in much more detail in new studies so that in the end there would be even more clarity as to which brain structures for hypnotic effects are indispensable for the treatment of pain and can best be modified by hypnosis.

## Author contributions

WM: Writing – original draft, Writing – review & editing. MF: Writing – original draft, Writing – review & editing. EN: Writing – original draft, Writing – review & editing.

## Footnotes

^1^Studies on the MEG are unfortunately missing in this review, as we could not identify empirical peer-reviewed articles on this topic.
